# Supplementary material for: A complex structure of escaping helium spanning more than half the orbit of the ultra-hot Jupiter WASP-121 b
Source: Nat Commun. 2025 Dec 8;16:10822. doi: 10.1038/s41467-025-66628-5 (PMC12686059; doi:10.1038/s41467-025-66628-5)
Supplement: Supplementary file 1 — Supplementary Information [file 41467_2025_66628_MOESM1_ESM.pdf]

Supplementary Information

A complex structure of escaping helium spanning more than half the orbit of the ultra-hot Jupiter WASP-121 b

Romain Allart[1*†], Louis-Philippe Coulombe[1], Yann Carteret[2], Jared Splinter[3,4], Lisa Dang[5,1], Vincent Bourrier[2], David Lafrenire[1], Loc Albert[1], tienne Artigau[1], Bjrn Benneke[6,1], Nicolas B. Cowan[3,4], Ren Doyon[1], Vigneshwaran Krishnamurthy[3], Ray Jayawardhana[7], Doug Johnstone[8,9], Adam B. Langeveld[7,10], Michael R. Meyer[11], Stefan Pelletier[2], Caroline Piaulet-Ghorayeb[12], Michael Radica[12], Jake Taylor[13], Jake D. Turner[10]

[1]Institut Trottier de recherche sur les exoplanètes, Département de Physique, Université de Montréal, Montréal, Québec, Canada.
[2]Observatoire astronomique de l'Université de Genève, 51 chemin Pegasi 1290 Versoix, Switzerland.
[3]Trottier Space Institute at McGill, 3550 rue University, Montréal, QC H3A 2A7, Canada.
[4]Department of Earth and Planetary Sciences, McGill University, 3450 rue University, Montréal, QC H3A OE8, Canada.
[5]Department of Physics and Astronomy, University of Waterloo, 200 University W, Waterloo, Ontario, Canada, N2L 3G1.
[6]Department of Earth, Planetary, and Space Sciences, University of California, Los Angeles, CA, USA.
[7]Department of Astronomy and Carl Sagan Institute, Cornell University, Ithaca, NY 14850, USA.
[8]NRC Herzberg Astronomy and Astrophysics, 5071 West Saanich Rd, Victoria, BC, V9E 2E7, Canada .
[9]Department of Physics and Astronomy, University of Victoria, Victoria, BC, V8P 5C2, Canada.
[10]Department of Physics and Astronomy, Johns Hopkins University, Baltimore, MD 21218, USA.
[11]Department of Astronomy, University of Michigan, 1085 S. University Ave,Ann Arbor, MI 48109, USA.
[12]Department of Astronomy & Astrophysics, University of Chicago, 5640 South Ellis Avenue, Chicago, IL 60637, USA.
[13]Department of Physics, University of Oxford, Parks Rd, Oxford, OX1 3PU, UK.

Contributing authors: romain.allart@umontreal.ca;
[†]SNSF Postdoctoral Fellow

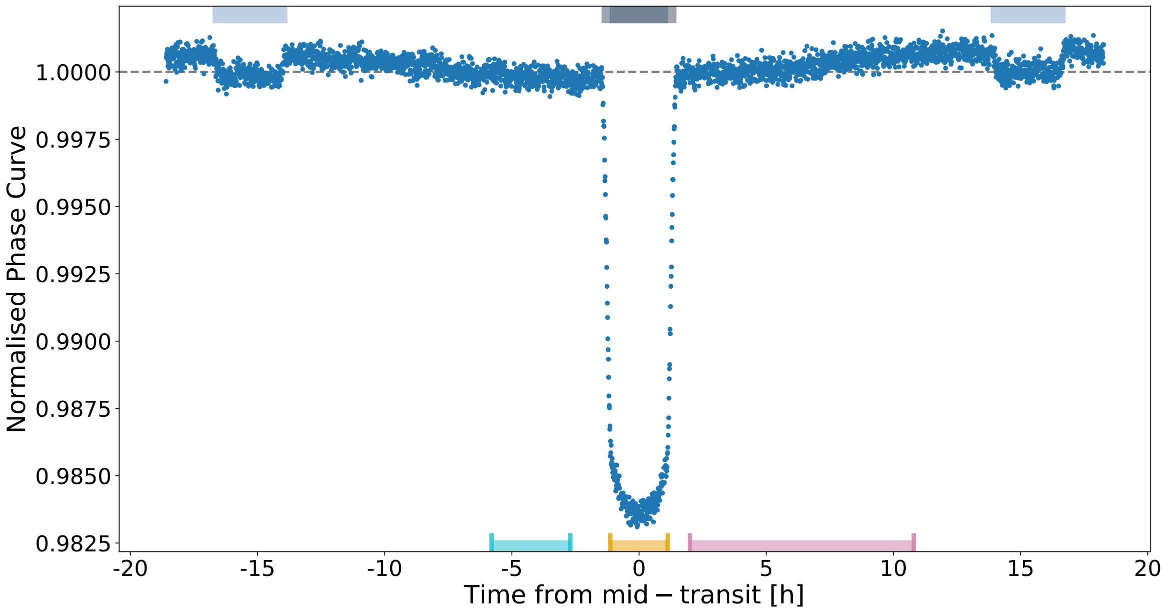

Supplementary Fig. 1: Reference white light phase curve. At the top, the two eclipses are indicated by the light blue gray regions, while the transit is shown by the grey regions with ingress and egress as the light grey region. At the bottom, the temporal signature is decomposed as the leading (cyan), transit (orange), and trailing signatures (pink), same colors as Fig. 3. Source data are provided as a Source Data file.

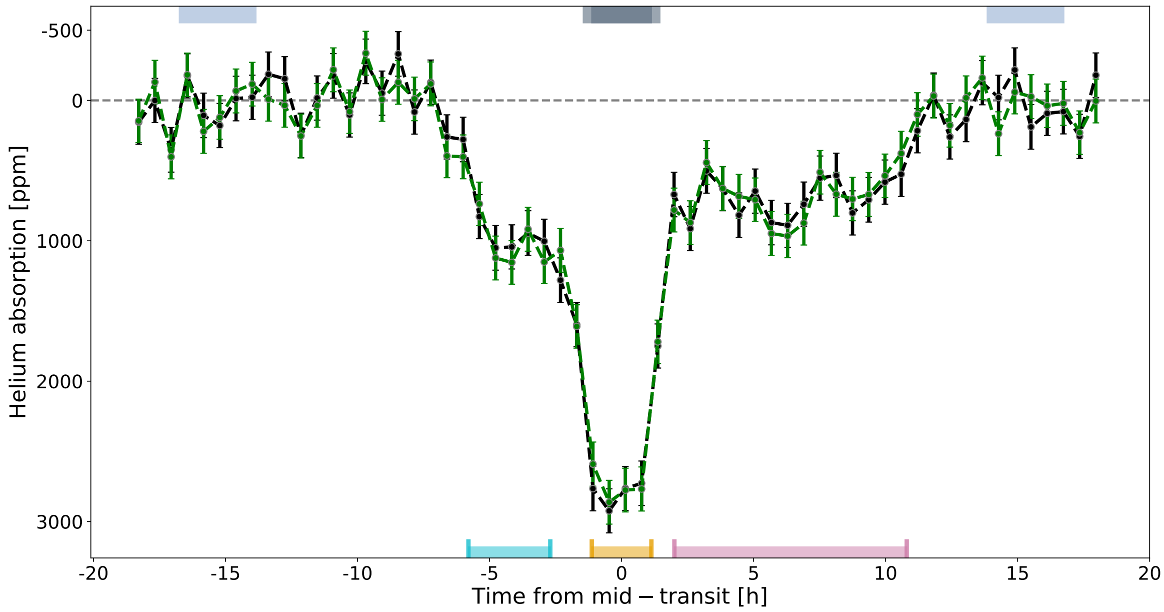

Supplementary Fig. 2: Excess absorption light curves (with 1σ error bars) of the helium spectral for the reduction with `NAMELESS` (black) and `exoTEDRF` (green). At the top, the two eclipses are indicated by the light blue gray regions, while the transit is shown by the grey regions with ingress and egress as the light gray region. At the bottom, the temporal signature is decomposed as the leading (cyan), transit (orange), and trailing signatures (pink), same colors as Fig. 3. Source data are provided as a Source Data file.

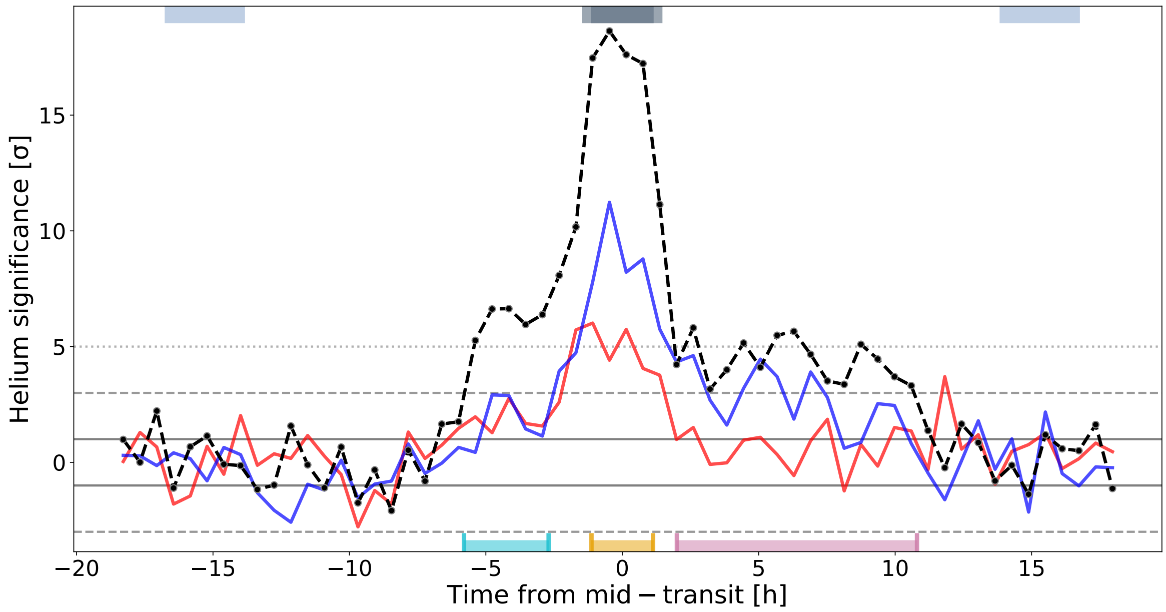

Supplementary Fig. 3: Helium detection significance as a function of time for the 60 temporal bins. The central pixel (10836.34 Å) is shown as the black dashed data points (with 1σ error bars). The two neighboring pixels of helium are shown in blue (10826.98 Å) and red (10845.70 Å). The horizontal grey curves are the $\pm 1\sigma$ thresholds, the horizontal dashed gray curves the $\pm 3\sigma$ thresholds, and the horizontal dotted gray line the $+5\sigma$ threshold. At the top, the two eclipses are indicated by the light blue gray regions, while the transit is shown by the gray regions with ingress and egress as the light gray region. At the bottom, the temporal signature is decomposed as the leading (cyan), transit (orange), and trailing signatures (pink), same colors as Fig. 3. Source data are provided as a Source Data file.

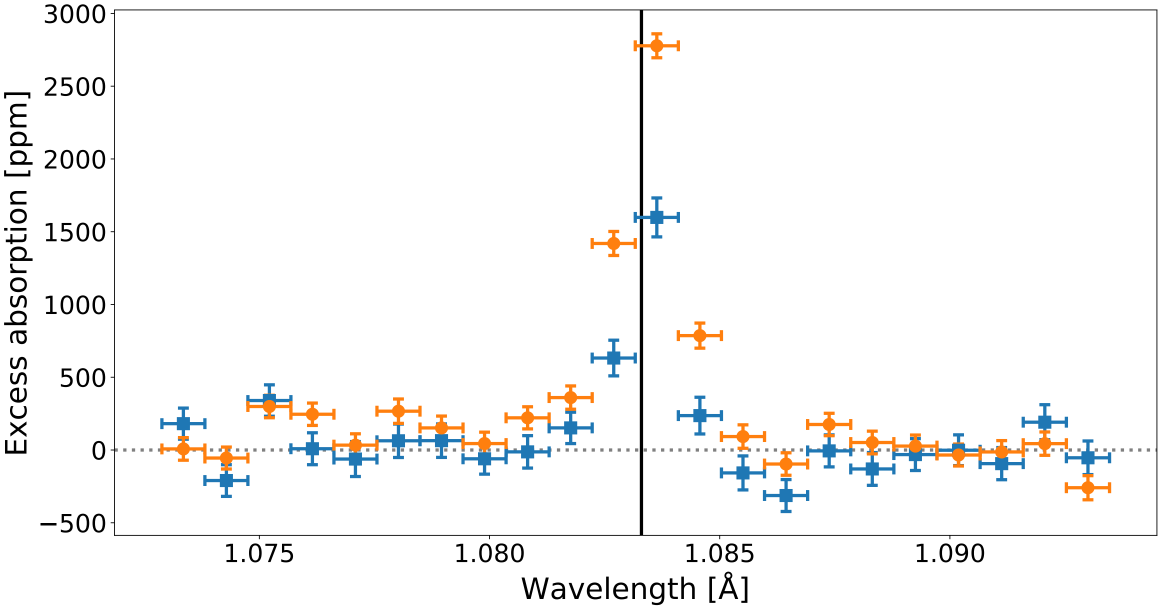

Supplementary Fig. 4: Comparison of the transmission spectrum (with 1σ error bars) around the helium triplet extracted during transit (orange) with the one extracted with a standard light curve fitting approach (blue). Source data are provided as a Source Data file.

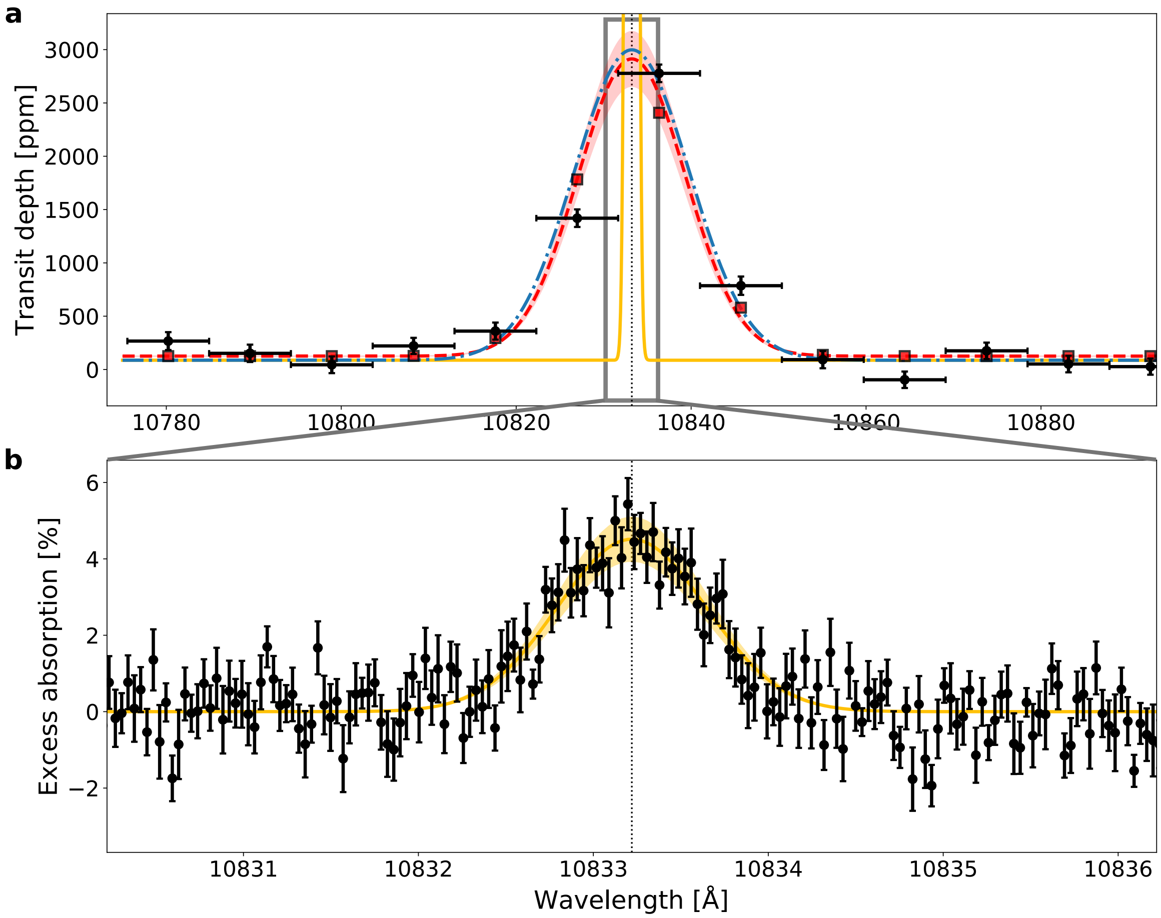

Supplementary Fig. 5: Simulated high-resolution transmission spectrum based on the JWST/NIRISS observations. Panel **a**: JWST transmission spectrum (with 1σ error bars) around the helium triplet with its best Gaussian fit in red. The red squares with black edges represent the best Gaussian fit binned to the JWST dataset. The blue Gaussian is the best fit of the high-resolution Gaussian with fixed width (yellow curve) after instrumental convolution. The grey box is inset of the bottom panel. Panel **b**: The best-fit high-resolution model is shown in yellow. The black data points are simulated datasets (with 1σ error bars) for a single NIRPS transit with the best-fit model injected in it. Source data are provided as a Source Data file.

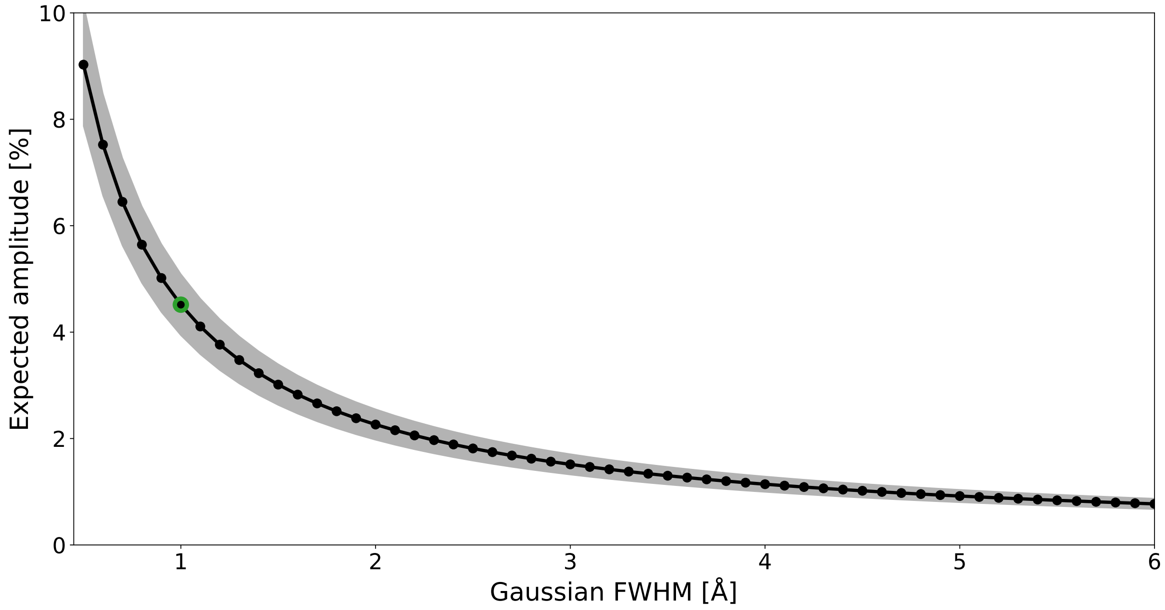

Supplementary Fig. 6: Variation of the expected amplitude of the high-resolution Gaussian profile as a function of its assumed FWHM, derived from the fit to the JWST datasets. Highlighted in green is the retrieved amplitude assuming an FWHM of 0.75 Å and presented in Supplementary Fig. 5. Source data are provided as a Source Data file.

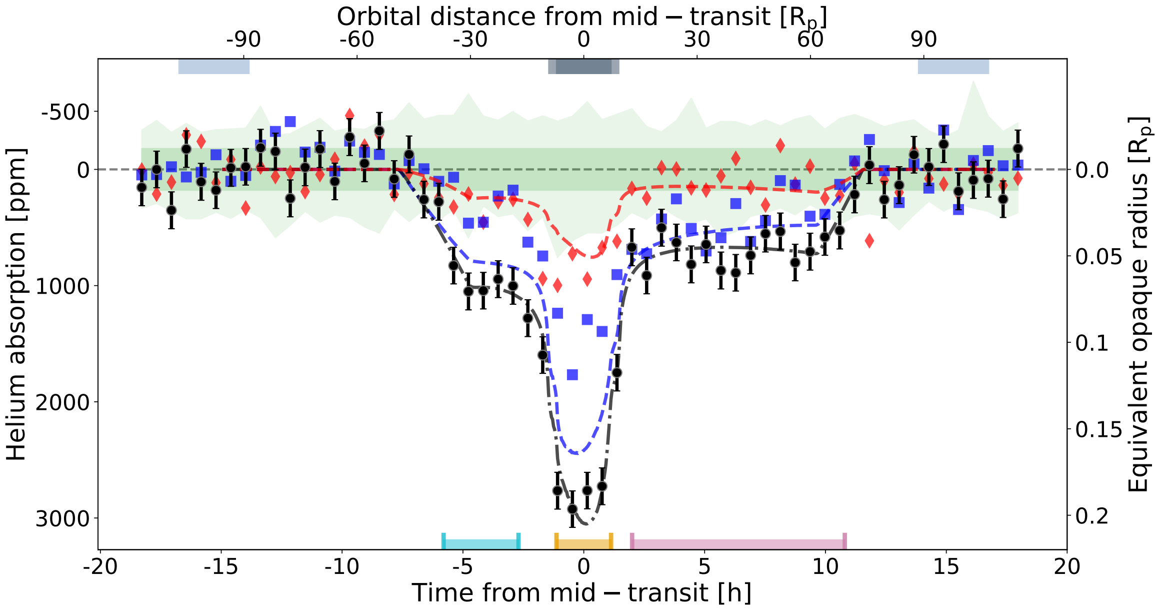

Supplementary Fig. 7: Comparison of the helium phase curve with our toy-model without the velocity gradient. The central pixel (10836.34 Å) is shown as the black dashed data points (with 1σ error bars). The two neighboring pixels of helium are shown in blue squares (10826.98 Å) and red diamonds (10845.70 Å). The dashed lines denote the best-fit toy model. The dark green region is the standard deviation of all the remaining pixels studied (from 10547.04 to 10714.83 Å and from 10958.12 to 11127.17 Å). In contrast, the light green region represents their upper and lower envelopes (obtained as the minimum and maximum flux value at each temporal bin). At the top, the two eclipses are indicated by the light blue gray regions, while the transit is shown by the grey regions with ingress and egress as the light grey regions. At the bottom, the temporal signature is decomposed as the leading (cyan), transit (orange), and trailing signatures (pink). Source data are provided as a Source Data file.

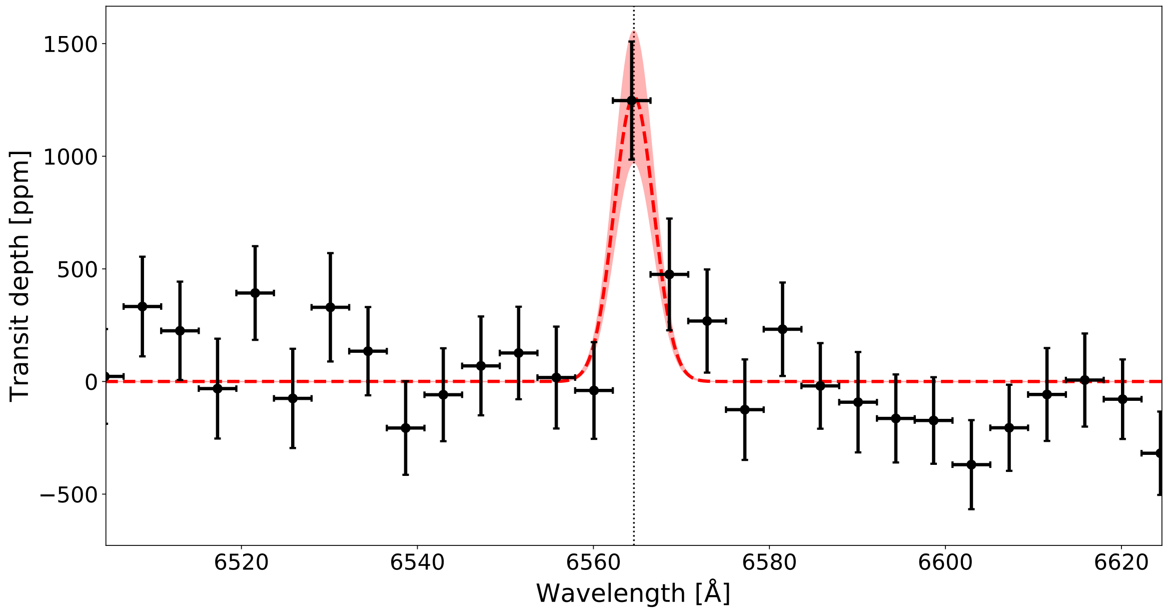

Supplementary Fig. 8: JWST transmission spectrum (with 1σ error bars) around the Hα line with its best Gaussian fit in red (amplitude of 1263$\pm$293 ppm). The transmission spectrum has been extracted with the standard light curve fitting approach. The Hα line position is indicated with the vertical black line. Source data are provided as a Source Data file.
